# NeuroD1 localizes to the presumptive ganglia and gut of the sea urchin larvae

**DOI:** 10.17912/micropub.biology.000682

**Published:** 2022-11-15

**Authors:** Kalin D. Konrad, Jia L. Song

**Affiliations:** 1 University of Delaware

## Abstract

NeuroD is a transcription factor (TF) that plays a dual role in vertebrate neurogenesis and glucose homeostasis in the pancreas. We identified a NeuroD antibody developed against human that cross-reacts with the sea urchin NeuroD1. NeuroD1 protein localizes to the presumptive ganglia and neurofilament structures in the ciliary band of the sea urchin larvae. In addition, we also observed NeuroD1 in the perinuclear region in the sea urchin gut which is analogous to the mammalian pancreas. These results suggest that NeuroD1 may play an evolutionarily conserved role in the invertebrate sea urchin.

**Figure 1. NeuroD1 may play an evolutionarily conserved role in the sea urchin embryo.**
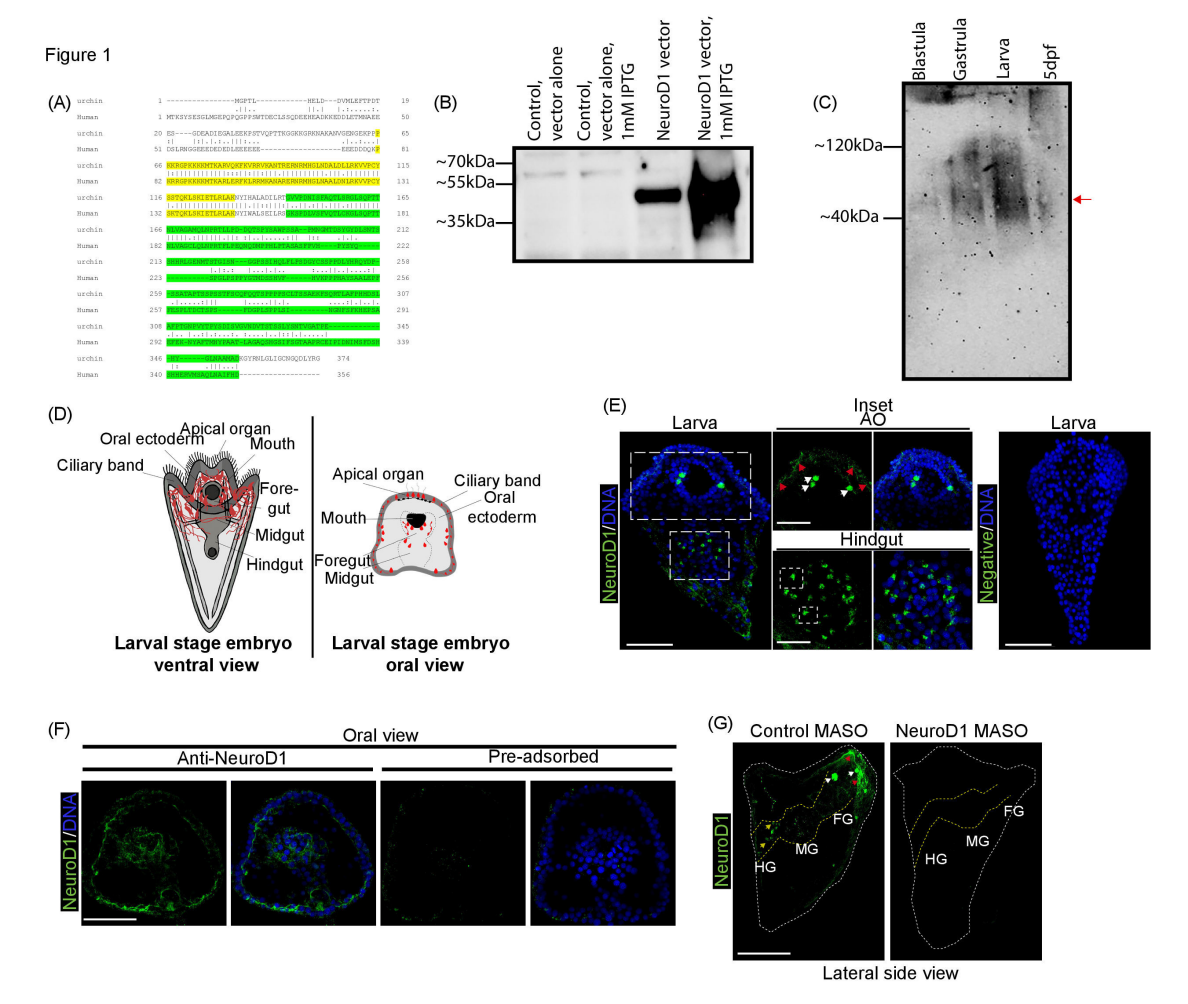
(A) The human NeuroD1 protein sequences is aligned with the sea urchin NeuroD1 protein using National Center for Biotechnology Information. SMART was used to identify DNA binding domain which is highlighted in yellow (Letunic and Bork 2018; Letunic et al. 2021). The antigenic region of the human NeuroD1 antibody is 31.4% identical to the sea urchin NeuroD1, highlighted in green. (B) Western blot analysis of NeuroD1 is depicted. Bacteria transformed with vector alone or NeuroD1 expression vector were cultured with or without IPTG. These bacterial lysates were collected and ran on an SDS-PAGE gel. Human NeuroD1 recognizes the sea urchin NeuroD1 recombinant protein, with the expected size of ~40-45kDa. (C) Western blot analysis of NeuroD1 indicates that NeuroD1 is expressed in various developmental stages of the sea urchin embryo. 5 dpf = 5 days post-fertilization. Arrow points to expected size of ~40-45kDa for NeuroD1. (D) Schematic of the different views of a sea urchin larva. (E) Sea urchin larvae were immunolabeled with NeuroD1 (green) and counterstained with DAPI to visualize DNA (blue). NeuroD1 is localized to presumptive ganglia (indicated by white arrows), neurofilaments (indicated by red arrows), and perinuclearly in cells of the gut (in dotted boxes). Five biological replicates. AO=apical organ. (F) The specificity of the NeuroD1 antibody is tested with pre-adsorption assay. Larvae were immunolabeled with pre-adsorbed or non-pre-adsorbed NeuroD1 antibody (green) and counterstained with DAPI (blue). (G) Embryos injected with NeuroD1 MASO had less NeuroD1 protein (green) compared to embryos injected with the control MASO, indicating antibody specificity and efficacy of the knockdown. Perinuclear localization of NeuroD1 in the gut (indicated by yellow arrows), presumptive ganglia (indicated by white arrows), neurofilaments (indicated by red arrows). The dotted yellow lines outline the gut, and the dotted white lines outline the ectoderm. FG=foregut, MG=midgut, and HG=hindgut. All scale bars correspond to 50 µm. 3 biological replicates for all the experiments unless specified otherwise. Maximum intensity projection of Z-stack confocal images is presented.

## Description

NeuroD (NeuroD1, NeuroD2, and NeuroD6) transcription factors (TFs) are members of the neuronal lineage basic helix-loop-helix family that regulate the transition from neuronal differentiation to maturation in vertebrate/invertebrate systems (Amador-Arjona et al. 2015; Aquino-Nunez et al. 2020; Cho and Tsai 2004; Huang et al. 2011; Liu et al. 2011; Masoudi et al. 2018; Matsuda et al. 2019; Pataskar et al. 2016). It also has an additional function in the pancreas where it regulates β cell glucose homeostasis (Gu et al. 2010; Itkin-Ansari et al. 2005; Perillo et al. 2018; Perillo et al. 2016). NeuroD1, specifically, is expressed early in mammalian embryos to regulate neurogenesis and β-cell development (Matsuda et al. 2019; Pataskar et al. 2016; Tutukova et al. 2021).

In the sea urchin embryo, *NeuroD1* transcript is expressed in the ectoderm on the anterior end of the embryo as well as some expression in the gastrula gut (48 hours post fertilization (hpf)) (Perillo et al. 2018). It has been previously proposed that the sea urchin larval gut contains pancreatic endocrine cells that may have co-opted some neuronal regulatory factors from an ancestral neuron (Perillo et al. 2018). During the larval stage (72 hpf), *NeuroD1* co-expresses with TFs in the ciliary band, such as neuronal differentiation factor, *Brn1/2/4,* and TFs with a pancreatic-like signature, such as *Neurogenin* and *Islet* (Perillo et al. 2018). In addition, it has been observed in mouse that NeuroD1 protein is localized in the intestine (Andrali et al. 2007; Li et al. 2019). The goal of this work is to identify a NeuroD1 antibody that cross-reacts with the sea urchin NeuroD1 and reveal its protein localization within the sea urchin embryo.

The NeuroD1 polyclonal antibody used for this study was developed against 197 amino acid sequence of the human NeuroD1 (total of 356 amino acids). This portion of the antigen excludes the DNA binding domain, since it’s a domain shared by all NeuroD family members (Berger et al. 2006; Grove et al. 2009; Reuter et al. 2022). We determined that human and sea urchin NeuroD1 proteins share an overall of 33.7% identify and their DNA binding domain share an 85.1% identity (Fig. 1A; DNA binding domain in yellow) (Letunic and Bork 2018; Letunic et al. 2021). Alignment of the human NeuroD1 antigenic sequence to that of the sea urchin NeuroD1 indicates a 31.4% identity (62 amino acids out of 197 amino acids aligned) (Fig. 1A highlighted in green).

We observed that the human NeuroD1 antibody was able to recognize the sea urchin NeuroD1 recombinant protein (Fig. 1B). The human NeuroD1 antibody recognizes a ~40-45kDa protein in sea urchin lysates from different developmental stages (Fig. 1C). This is close to the predicted size of the sea urchin NeuroD1 at 42kDa (Stothard 2000). In addition, we observed that the strongest expression of NeuroD1 was during the larval stage (72hpf) (Fig. 1C). The ~40-45kDa size of the detected NeuroD1 protein is similar to the predicted size of human NeuroD1 at 40kDa and what has been observed on westerns blot using mouse and human cell lysates (Andrali et al. 2007; Coordinators 2013; Gao et al. 2011; Kitazono et al. 2020).

Using this human NeuroD1 antibody, we determined that NeuroD1 is expressed in three cells where the presumptive ganglia are in the mouth region, similar to where *NeuroD1* transcripts were observed (Paganos et al. 2021; Perillo et al. 2018). We also observed neurofilament structures in the ciliary band where neurons reside within the sea urchin larva (Figs. 1D-E). Based on prior immunolabeling with Synaptotagmin B (SynB), which is part of the SNARE family mediating synaptic release of neurotransmitters, NeuroD1 seems to be expressed where SynB is localized (Burke et al. 2006; DeBello et al. 1993; Konrad and Song 2022). However, without co-immunolabel NeuroD1 with a neurofilament antibody, we cannot conclude that these are neurofilaments. Even though NeuroD1 is a TF, it has been previously observed that mouse NeuroD1 protein is also diffusely expressed in the cytoplasm in pancreatic cells as well as in the cytoplasm of neurons (Andrali et al. 2007; Armelloni et al. 2018; Gu et al. 2010; Puligilla et al. 2010). In the pancreatic beta-cell line (MIN6), NeuroD1 is normally perinuclearly localized (Andrali et al. 2007). In response to glucose, NeuroD1 enters the nucleus to activate insulin expression, to maintain homeostasis in the kidneys (Andrali et al. 2007; Armelloni et al. 2018). This may explain our observation of NeuroD1 in the perinuclear region in the sea urchin gut cells which would be analogous to the mammalian pancreas, suggesting that NeuroD1 may act like a pancreatic-like TF similar to the vertebrate NeuroD1 (Gu et al. 2010; Perillo et al. 2018; Perillo et al. 2016).

To determine the specificity of the NeuroD1 antibody, we performed a pre-adsorption assay. Pre-adsorption of the human NeuroD1 antibody with or without bacterial lysate expressing the sea urchin NeuroD1 recombinant protein was used in immunolabeling experiments (Fig. 1F). We observed that pre-adsorbed NeuroD1 antibody failed to detect the presumptive ganglia and neurofilamentous structures in sea urchin larvae, compared to the non-pre-adsorbed NeuroD1 antibody (Fig. 1F). These data indicate that the human NeuroD1 antibody specifically recognizes sea urchin structures expressing NeuroD1.

To further test the specificity of the antibody, we performed loss-of-function studies of NeuroD1 with a translation-blocking morpholino (NeuroD1 MASO) (Fig. 1G). Zygotes were microinjected with control MASO or NeuroD1 MASO and incubated to the larval stage. Results indicate that NeuroD1 protein was reduced in NeuroD1 MASO-injected embryos compared to the control MASO-injected embryos. Specifically, we did not observe the neurofilament structures in the ciliary band (indicated by red arrows), the ganglia structures (indicated by white arrows), and the perinuclear localization of NeuroD1 in the larval gut (indicated by yellow arrows) in the NeuroD1 MASO-injected embryos (Fig. 1G), indicating the efficacy of NeuroD1 knockdown and specificity of the NeuroD1 antibody.

We have previously observed that NeuroD1 knockdown larvae have decreased expression of *SoxC* (neural progenitor gene) *,* *Delta* (neural differentiation gene), and, *Elav* (RNA-binding protein gene important for neuronal plasticity) (Konrad and Song 2022). Therefore, NeuroD1 performs a similar function during sea urchin neurogenesis compared to other systems and further supports the idea that its function is evolutionarily conserved (Cho and Tsai 2004; Kamath et al. 2005; Konrad and Song 2022; Singh et al. 2022).

Overall, we observed that NeuroD1 is expressed in the presumptive ganglia, neurofilament and pancreatic-like cells of the sea urchin embryo, similar to immunostaining patterns observed in other vertebrate systems (Armelloni et al. 2018; Boutin et al. 2010; Gu et al. 2010; Kamath et al. 2005; Puligilla et al. 2010). Even though the sea urchin nervous system and gut (that expresses pancreatic-like TFs) are vastly different from the vertebrate system, we can conclude that the NeuroD1 expression patterns share striking similarities between the sea urchin and mammalian systems. In addition, we demonstrate that the antibody developed against the human NeuroD1 cross-reacts with the sea urchin NeuroD1 protein, and therefore, can be used as a tool to understand its evolutionarily conserved functions in other systems.

## Methods

***Animals***

Adult *Strongylocentrotus purpuratus* were collected from the California coast (Pt. Loma Marine Invertebrate Lab or Marinus Scientific, LLC.). Adult animals and embryonic cultures were incubated in artificial sea water or filtered natural sea water, respectively, at 15°C. The natural sea water is collected from the Indian River Inlet (University of Delaware).

***Protein analysis and alignment***

Using Clustal Omega, we aligned the protein sequences from the sea urchin and human NeuroD1. SMART was used to identify DNA binding domain (Letunic and Bork 2018; Letunic et al. 2021). Using the National Center for Biotechnology Information protein alignment, we obtained the percentages of the alignment (Altschul et al. 1990; Coordinators 2013).

***Western blot***

To test if the NeuroD1 antibody developed against the human antigen would cross-react with the sea urchin NeuroD1, we first cloned the sea urchin NeuroD1 into an expression vector pNOTAT (Nagahara et al. 1998) using forward primer: 5’ GGATCC ATGGGCCCCACCCTACATGA 3’ and reverse primer: 5’ CTCGAG TTAACCGCGGTATAAATCTTGTC 3’. Forward primer contains a BamHI restriction site (underlined), and the reverse primer contains XhoI restriction site (underlined). Plasmids cloned with NeuroD1 were validated through DNA sequencing (University of Delaware DNA Sequencing and Genotyping Center, Newark, DE and Genewiz, South Plainfield, NJ). NeuroD1-pNOTAT was transformed into C41 competent cells (Lucigen, Middleton, WI) and induced with IPTG at 1mM for protein expression. Lysates from bacteria transformed with pNOTAT vector (negative control) and lysates from bacteria transformed with the sea urchin NeuroD1 with or without 1mM IPTG induction were run on a 12% SDS-PAGE gel made by following instructions from the manufacturer (BioRad, Philadelphia, PA). The protein gel was transferred to the PVDF membrane (BioRad, Philadelphia, PA) using a wet transfer system (BioRad, Philadelphia, PA). The blot was blocked in 3% bovine serum albumin (BSA) (Research Products International, Mount Prospect, IL) in TBST (Tris 50 mM, pH 7.5, NaCl 184 mM, Tween-20 0.05%) (Fisher Chemical, Hampton, NH) for 1 hour at room temperature.

For the sea urchin lysate, we collected embryos at different developmental stages. The embryos were lysed using a Tris- based buffer containing, Tris 75mM, pH 7.8, NaCl 150mM (Fisher Chemical, Hampton, NH), and 1x protease inhibitor (Promega, Madison, WI). Total protein was measured using Bradford protein assay and obtaining the absorbance using biophotometer (Eppendorf, Enfield, CT). 5µg of total protein from each developmental stage was loaded in each well of the SDS-PAGE gel. Then NeuroD1 antibody at 1:1,000 (ABclonal company cat# A1147, Woburn, MA) was incubated in 3% BSA in TBST overnight at 4°C. The blot was exposed with SuperSignal West Pico Chemiluminescent Substrate (Thermo Fisher Scientific, Waltham, MA) and imaged with BioRad Chemidoc Gel Imager (BioRad, Philadelphia, PA). We used Pageruler Multicolor ladder (Thermo Fisher Scientific, Waltham, MA) for the western blot with the bacterial lysate (Fig. 1B), and we used MagicMark ladder (Thermo Fisher Scientific, Waltham, MA) for the time course western blot (Fig. 1C).

***Immunolabeling of NeuroD1***

Larval stage (72hpf) embryos were fixed in 4% paraformaldehyde for 10 minutes and post fixed with 100% acetone for 1 minute at room temperature. Larvae were washed with 1X phosphate buffer saline (PBS) (BioRad, Philadelphia, PA) PBST containing 0.1% Triton X-100 (Thermo Fisher Scientific, Waltham, MA) and incubated with the NeuroD1 antibody at 1:250 (ABclonal company cat# A1147, Woburn, MA) for two nights at 4°C in blocking buffer (10% Bovine serum albumin (MilliporeSigma, St. Louis, MO) in PBST-0.1% Triton X-100). Embryos were washed three times with PBST and incubated with goat anti-rabbit Alexa 488 at 1:300 in blocking buffer for 1 hour at room temperature (Thermo Fisher Scientific, Waltham, MA). Embryos were counterstained with DAPI (NucBlue; Thermo Fisher Scientific, Waltham, MA). Images were taken using the Zeiss LSM 880 scanning confocal microscope (Carl Zeiss Incorporation, White Plains, NY). The maximum intensity projections of Z-stack images were acquired with Zen software and processed with Adobe Photoshop and Adobe Illustrator (Adobe, San Jose, CA).

***Pre-adsorption assay of NeuroD1***

A pre-adsorption assay was performed to test the specificity of the NeuroD1 antibody, by incubating the NeuroD1 antibody with bacterial lysate expressing the sea urchin NeuroD1 protein. First, 50 µL of prepared bacterial lysate was incubated with the PVDF (BioRad, Philadelphia, PA), membranes (0.5 cm x 0.5 cm) in Eppendorf tubes and rocked for two nights at 4°C. Then the NeuroD1 antibody was added at 1:250 in blocking buffer (PBST- 0.1% Triton in 4% sheep serum) and incubated with the PVDF containing the blocking buffer or the sea urchin NeuroD1 for two nights at 4°C. NeuroD1 antibody pre-adsorbed with bacterial lysate with or without sea urchin NeuroD1 was used in immunolabeling experiments. Embryos were washed with PBST and exposed with goat anti-rabbit 488 in blocking buffer for 1 hour at room temperature counterstained with DAPI. Images were acquired with Zeiss LSM 880 scanning confocal microscope (Carl Zeiss Incorporation, White Plains, NY).

***Microinjections***

Injection solutions contained 20% sterile glycerol, 2 mg/mL 10,000 MW FITC lysine charged dextran (ThermoFisher Scientific, Waltham, MA), and 30µM of NeuroD1 MASO. The sequence of NeuroD1 MASO is: 5’ AGTTCCTTTTTTATGACGTT 3’ (Gene Tools, LLC, Philomath, Oregon). The NeuroD1 MASO was blasted against the sea urchin genome and no other targets are identified, indicating the specificity of the NeuroD1 MASO. The negative control MASO was designed against human *beta-globin* intron and its sequence is: 5’CCTCTTACCTCAGTTACAATTTATA 3’ (Gene Tools, LLC, Philomath, Oregon). Microinjections were performed as previously described (Konrad and Song 2022; Stepicheva and Song 2014).
